# Secondary contact rather than coexistence—*Erebia* butterflies in the Alps

**DOI:** 10.1111/evo.14615

**Published:** 2022-10-05

**Authors:** Hannah Augustijnen, Theofania Patsiou, Kay Lucek

**Affiliations:** ^1^ Department of Environmental Sciences University of Basel Basel CH‐4056 Switzerland; ^2^ Institute of Plant Sciences University of Bern Bern CH‐3013 Switzerland; ^3^ Department of Biology University of Fribourg Fribourg CH‐1700 Switzerland

**Keywords:** Lepidoptera, **P** matrix, reinforcement, secondary contact

## Abstract

Secondary contact zones are ideal systems to study the processes that govern the evolution of reproductive barriers, especially at advanced stages of the speciation process. An increase in reproductive isolation resulting from selection against maladaptive hybrids is thought to contribute to reproductive barrier buildup in secondary contact zones. Although such processes have been invoked for many systems, it remains unclear to which extent they influence contact zone dynamics in nature. Here, we study a very narrow contact zone between the butterfly species *Erebia cassioides* and *Erebia tyndarus* in the Swiss Alps. We quantified phenotypic traits related to wing shape and reproduction as well as ecology to compare the degree of intra‐ and interspecific differentiation. Even though only very few first‐generation hybrids occur, we find no strong indications for current reinforcing selection, suggesting that if reinforcement occurred in our system, it likely operated in the past. Additionally, we show that both species differ less in their ecological niche at the contact zone than elsewhere, which could explain why coexistence between these butterflies may currently not be possible.

When closely related lineages become geographically isolated, they may accumulate genetic incompatibilities through drift and divergent selection over time (Turelli et al. 2001; Coyne and Orr 2004). The evolutionary consequence of secondary contact between such lineages depends on the presence and strength of reproductive barriers that evolved during allopatry (Butlin et al. 2012; Canestrelli et al. 2016). Outcomes can range from substantial admixture when barriers are weak, to the formation of hybrid zones whose widths depend on the strengths of the barriers involved, and eventually to coexistence without gene flow (Harrison and Larson 2014; Gompert et al. 2017). Secondary contact zones provide excellent opportunities to investigate the evolution and interaction of reproductive barriers, often at an advanced stage of the speciation process (Gompert et al. 2017; Kulmuni et al. 2020). The reason is that selection against hybrids upon secondary contact could trigger the evolution of additional barriers to gene flow through reinforcement, that is, the evolutionary process by which reproductive isolation increases in response to costly hybridization (Dobzhansky 1940; Servedio and Noor 2003; Butlin and Smadja 2018). Although reinforcement has been invoked for some systems (e.g., Hoskin et al. 2005; Kronforst et al. 2007; Hopkins and Rausher, 2012; Turelli et al. 2014), the relative frequency and importance of this process in nature remain debated (Matute and Cooper 2021). Expanding on findings from a former study (Lucek et al. 2020), we investigated the outcome of secondary contact between two sibling butterfly species of the genus *Erebia* from the Swiss Alps that form a very narrow contact zone (<500 m) and assessed the potential for phenotypic signatures of reinforcement.

Reinforcement is predicted to be associated with the evolution of prezygotic barriers and may result in increased interspecific phenotypic differentiation in a zone of secondary contact compared to allopatric sites (Coyne and Orr 2004; Servedio 2009, but see Butlin and Ritchie 2013). Such character displacement often results in phenotypic and genetic clines across the contact zone, and in cases of resource competition or niche segregation, ecological clines may similarly occur (Barton and Hewitt 1985; Goldberg and Lande 2006; Gompert et al. 2017). The drivers of prezygotic isolation and the associated traits that experience selection often differ between taxa (Ravinet et al. 2017; Merot et al. 2017). If prezygotic isolation involves mate choice, divergence in mating relevant traits may be reinforced upon secondary contact, leading to reproductive character displacement (RCD; Gröning and Hochkirch 2008; Pfennig and Pfennig 2009). RCD has been shown for advertisement calls in chorus frogs (*Pseudacris* sp.; Lemmon and Lemmon, 2010) or color patterning of butterfly wings (Hinojosa et al. 2020). Divergence in genital morphology has similarly been invoked to result in RCD (Hollander et al., 2013). The latter may be especially true for organisms with internal fertilization, such as insects, where lock‐and‐key mechanisms have been suggested to be a powerful agent of selection against hybrids (Sota and Kubota 1998). For example, increased difference in the lengths of the male copulatory organ upon secondary contact has been shown to lead to failure of heterospecific matings in carabid beetles (*Carabus* sp.), resulting in the evolution of increased premating isolation (Usami et al. 2006; Nishimura et al. 2022). Importantly, character displacement resulting from reinforcement may increase trait divergence in either one of the two species involved, or in both (Cooley 2007; Wheatcroft and Qvarnström, 2017; Dyer et al. 2018).

Secondary contact and reinforcement have been suggested to affect the multivariate phenotypic covariance structure (Blows and Higgie 2003; Dochtermann and Matocq 2016). Multivariate phenotypic evolution is thought to be constrained along so‐called “lines of least resistance,” that is, the leading eigenvector of the **G** matrix (***g***_max_), which summarizes the additive genetic variances and covariances (Lande 1979; Lande and Arnold 1983; Schluter 1996; Steppan et al. 2002). Biologically, this axis captures the largest fraction of the genetic variance and is predicted to be shaped by selection and drift (Lande and Arnold 1983; Steppan et al. 2002; Marroig and Cheverud 2005; Arnold et al. 2008). In the absence of quantitative genetic data, the **G** matrix may be surrogated by the **P** matrix and ***p***_max_, based on phenotypic data from wild populations (Cheverud 1988). This method is valid when phenotypic traits are heritable (Lande 1979), as has been found for many taxa (Cheverud 1988; Leinonen et al. 2011), including butterfly wing patterns (e.g., Palmer and Kronforst 2020; Nadeau et al. 2016) or insect genital morphology (e.g., Higgins et al. 2009; Andrade et al. 2009). Different **P** matrices can be compared by calculating the angle *θ* between different ***p***_max_ (Schluter 1996). Although the effects of gene flow and hybridization on the **G**/**P** matrices have been studied both from a theoretical and empirical perspective (e.g., Guillaume and Whitlock 2007; Lucek et al., 2014a; Lucek et al. 2017), few empirical studies have looked at the outcome of secondary contact on the **G**/**P** matrices (Blows and Higgie 2003; Dochtermann and Matocq 2016).

*Erebia* is a genus of cold‐adapted butterflies (Sonderegger 2005; Peña et al. 2015). The diversification of *Erebia* has been associated with differentiation in distinct glacial refugia due to the Quaternary glacial cycles (Sonderegger 2005; Schmitt et al. 2006; Schmitt and Haubrich 2008; Albre et al. 2008; Schmitt et al. 2014). Following postglacial range expansions, distantly related *Erebia* species often coexist and exploit different microhabitats (Kleckova et al. 2014). However, closely related species or lineages exclude each other in several cases by forming very narrow secondary contact zones (Schmitt and Müller 2007; Descimon and Mallet 2009; Cupedo, 2007; Lucek et al. 2020). Given the abundance of contact zones between different *Erebia* species or lineages, they provide an excellent system to study the outcome of secondary contact. For example, *Erebia cassioides* and *Erebia tyndarus*, two evolutionarily young species that split about 2 million years ago (Peña et al. 2015), recolonized the Alps from different refugia (Schmitt et al. 2016; Gratton et al. 2016, Lucek et al. 2020), and form a very narrow contact zone in the central Alps, which has been stable since at least the 1950s (Warren 1954; Sonderegger 2005). A preliminary study with few individuals found that the two closely related species form a very narrow genomic cline and found only a few F_1_ hybrid individuals, suggesting selection against interspecific gene flow in this system (Lucek et al. 2020). The genomic cline overlapped with the presence/absence of the endosymbiotic bacterium *Wolbachia*, where 90% of *E. cassioides* were infected, as were the F_1_ hybrids, whereas none of the studied *E. tyndarus* carried the symbiont (Lucek et al. 2020). Although the potential role of *Wolbachia* in *Erebia* is still unknown (Lucek et al. 2021), it may act as an intrinsic postzygotic barrier to gene flow, potentially causing sterility of hybrids as in other butterflies (Nice et al. 2009). Indeed, *E. tyndarus* can be crossed with moderate success with *E. cassioides* when for the latter a distinct lineage from the Eastern Alps is used (Lorkovic 1958) that shares a *Wolbachia* strain with nearby *E. tyndarus* populations (Lucek et al. 2021). The genomic cline also overlapped with a phenotypic cline on wing patterns (Lucek et al. 2020). Wing‐pattern recognition is often related to mate choice in butterflies (e.g., Kemp and Rutowski 2011; Hinojosa et al. 2020). RCD could thus have evolved to avoid costly hybridization. However, Lucek et al. (2020) could not test this, as allopatric populations needed for comparison were unavailable.

Here, we expand on the study of Lucek et al. (2020) and assess the potential footprint of reinforcement upon secondary contact, that is, evidence for RCD on male genital morphology and wing shape. In a first step, we quantify the degree of intra‐ and interspecific phenotypic differentiation between individuals from geographically distant allopatric sites and expand on the formerly described contact zone in terms of sampling and geographical extent. Under RCD, we predicted increased phenotypic differentiation in the secondary contact zone compared to the degree of allopatric differentiation in one or both species. We further compared the degree of multivariate phenotypic differentiation using **P** matrices, similarly predicting a shift in the multivariate phenotype between individuals from the contact zone and the allopatric sites or along the secondary contact zone. Finally, we also test for ecological character displacement along the contact zone of *E. cassioides* and *E. tyndarus*.

## Materials and Methods

### SAMPLING AND DATA COLLECTION

**Figure 1:**
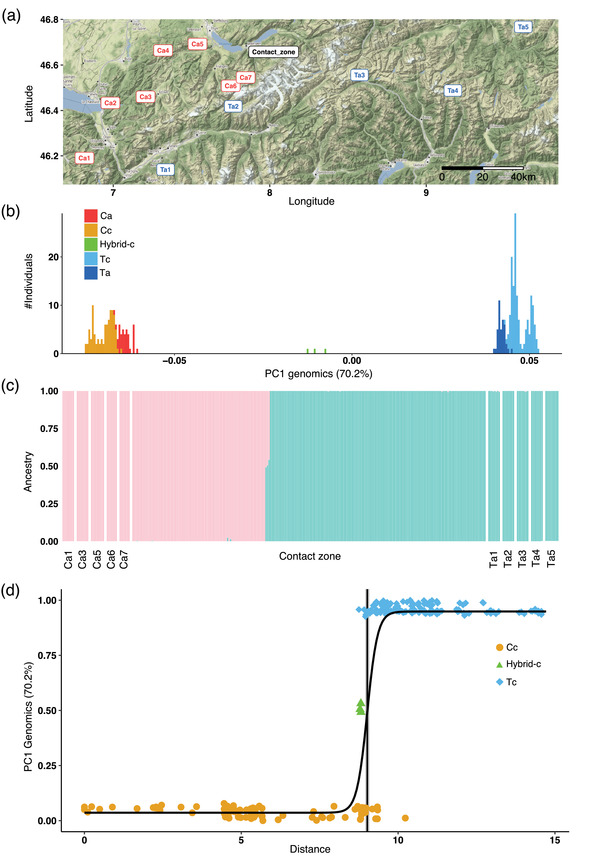
Sampling locations and genomic ancestry of *Erebia cassioides* and *Erebia tyndarus*. (a) Map depicting sampling locations across Switzerland (Source: Google Maps, 2021; see Table S1). (b, c) Genetic assignment of *E. cassioides* and *E. tyndarus*. (b) Barplot of the first principal component (PC) axis based on 2387 SNPs. Red = allopatric *E. cassioides* (Ca), orange = contact zone *E. cassioides* (Cc), green = hybrids, dark blue = allopatric *E. tyndarus* (Ta), light blue = contact zone *E. tyndarus* (Tc). (c) ADMIXTURE result for *K* = 2. (d) Cline fit for scaled scores of the genomic PC1 along a west‐east transect in the contact zone. The black line represents the fitted cline and the vertical black bar the cline center with its 95% confidence interval in gray.

We collected 841 male specimens of *E. cassioides* and *E. tyndarus* from 13 sites across Switzerland between June and September 2017–2020, with the vast majority caught in August (Figs. 1a, S1; Table S1). All individuals were caught by hand‐netting and immediately euthanized with an overdose of ethyl acetate. For each specimen, we recorded its place‐of‐catch (GPS). We clipped the wings of each specimen, kept them in paper bags for further morphological analyses, and stored the body at –20°C. We used the coordinates to retrieve abiotic environmental parameters from a 25‐m‐resolution climatic dataset for Switzerland (Broennimann 2018). Monthly data on mean, minimum, and maximum temperature (°C), precipitation (mm), growing degree days (i.e., the accumulation of temperature units during days where the temperature is above the 0°C threshold for alpine plant growth), and potential evapotranspiration (mm/day) were extracted for July‐September, the months of active flight for *E. cassioides* and *E. tyndarus* (Sonderegger 2005). We further extracted the 19 bioclim variables as designated in the Worldclim database for the same time period (Fick and Hijmans 2017). Finally, because the geological substrate is often associated with broad‐scale species distributions of mountain butterflies, as it may be related to the presence of food plants (Sonderegger 2005; Illán et al. 2010), we extracted substrate information for each specimen from the EuroGeoSurvey European Geological Data Infrastructure (Tulstrup et al. 2016).

Intra‐ and interspecific phenotypic differences in wing morphology, especially in the shape and extent of the orange spot on the dorsal surface of the forewing, have been observed for various *Erebia* species in the Swiss Alps (Sonderegger 2005). We assessed the phenotypic variation of our samples by digitizing the right dorsal surface of the forewings of 833 out of 841 specimens with a flatbed scanner. Eight samples were discarded due to insufficient wing quality. When damaged, we scanned the left dorsal surface of the forewing instead, and the image was flipped horizontally (*N* = 127). We captured phenotypic variation among and within *Erebia* populations by placing 23 landmarks on scanned wing images, focusing on defining wing shape based on venation and the shape and extent of the orange spot (Fig. S2a). Landmarks were placed using TPSDIG2 2.31 (Rohlf 2015), and Procrustes coordinates were calculated in morphoj 1.07a (Klingenberg 2011). Additionally, we measured wing length, defined as the distance between landmarks 1 and 4 (Fig. S2a), using ImageJ 1.53a (Abràmoff et al. 2004). We applied a size correction by taking the regression residuals of the untransformed traits against measured wing length for each individual.

Like wing morphology, male genital morphology is a common characteristic to distinguish between *Erebia* species and subspecies (Sonderegger 2005; Cupedo 2014). Therefore, we clipped the genital apparatus of all 833 specimens used for wing morphology before macerating them in a 13% sodium hydroxide solution at room temperature for 24 h. Forceps were used to remove additional tissue. We stained the remaining sclerotized genitalia with a 3% Eosin Y solution in 70% ethanol for 5 min. Subsequently, we washed the genitalia in 70% ethanol, once for 5 min and then for 20 min. Stained genitalia were stored in 90% ethanol at –20°C. We photographed the valve structures using a stereomicroscope (Leica M205 C, Leica Microsystems, Wetzlar, Germany). We placed 11 landmarks on each resulting image with TPSDIG2, covering valve length and shape and shape and positioning of the first three‐valve teeth (Fig. S2b), reflecting traits known to differ between *E. cassioides* and *E. tyndarus* (Sonderegger 2005).

### GENOTYPING AND SPECIES ASSIGNMENT

To assess the extent of interspecific gene flow, we used the restriction site‐associated DNA (RAD) sequence data from Lucek et al. (2020; *N* = 84; NCBI BioProject Accession: PRJNA640280) and combined it with information from 235 newly genotyped individuals. Of the latter, 152 were collected along the contact zone and the remainder across 10 allopatric sites (Fig. 1a). DNA extraction followed Lucek et al. (2020). We used whole‐genome resequencing (WGS) on a single Illumina NovaSeq 6000 S4 flow cell. Library preparation and sequencing were outsourced to the Genomics Facility Basel (D‐BSSE of ETH Zurich). We aligned all data against the *Erebia ligea* genome (NCBI GCA_917051295.2) using BWA mem version 0.7.17 (Li 2013), followed by genotyping with BCFtools version 1.15 (Li 2011). Only sites covered by both the RAD and the WGS datasets were retained. We then used VCFtools version 0.1.16 (Danecek et al., 2011) to apply a filter for minor allele frequencies (MAF) ≥0.04, remove indels, and to remove SNPs that were non‐biallelic, had a genotype quality score ≤20, had >60% missing data across all samples, or had a depth <5 or >30. Our filtering resulted in 2387 SNPs for a total of 319 individuals. To perform a principal component (PC) analysis, we used Plink version 2 (Chang et al., 2015). We further ran ADMIXTURE version 1.3.0 (Alexander et al. 2009) assuming two genetic clusters (*K* = 2) to test for interspecific gene flow.

*Erebia cassioides* and *E. tyndarus* can be challenging to distinguish in the field, particularly upon secondary contact, where potential hybrids may show intermediate phenotypes (Sonderegger 2005; Lucek et al. 2020). We consequently employed a linear discriminant analysis (LDA) including all morphological traits for both wing and genital shapes to assign the individuals from the contact zone that were not genotyped to either species. To estimate the reliability of this approach, we first conducted an LDA using all allopatric individuals (*N* = 347) and all genotyped males from the contact zone from Lucek et al. (2021, 2020) (*N* = 42). We then assigned all remaining individuals from the contact zone (*N* = 444) to either species with the *predict* function in R version 3.6.3 (R Core Team 2020). This initial prediction was validated based on the 152 newly genotyped individuals from the contact zone. Of these, 97% were correctly assigned by the initial LDA to their respective species. Finally, we repeated our LDA assignment using all genotyped individuals, and to reduce potential false‐positive assignments, we included only individuals within the 97% confidence interval (CI) for the respective phenotypic traits studied in all subsequent analyses. Given the low frequency of hybrids (*N* = 3; Fig. 1), and their phenotypically intermediate characters (Fig. S3), we excluded these from subsequent phenotypic analyses.

### PHENOTYPIC DIFFERENTIATION BETWEEN ALLOPATRIC SITES

To test if individuals from the contact zones would differ phenotypically from allopatric sites, we first summarized the phenotypic variation across all individuals with PC analyses for wing shape, orange spot, and genital shape. We then tested for intra‐ and interspecific morphological differences between allopatric individuals and individuals from the contact zone by fitting linear mixed‐effects models with lme4 (Bates et al. 2015) in R on the PC1 scores for wing shape, orange spot, and genital shape, with *species* and *population type* (i.e., allopatric or contact zone) as explanatory variables. We included location as a random effect and estimated the significance of each model with a type II Wald *χ* ^2^ test.

To capture intra‐ and interspecific changes in the phenotypic variance‐covariance structure, we also compared the phenotypic covariance matrices (**P** matrices) among populations, by first calculating the angles (*θ*) between their leading eigenvectors ***p***_max_ and second, the pairwise Mahalanobis distances between **P** matrices. For *θ*, we calculated the dot product's inversed cosine between two ***p***_max_ divided by the summed length of both ***p***_max_ (Schluter 1996). To establish the statistical significance of each comparison, we used 10,000 bootstrap replicates as implemented in Lucek et al. 2014a and 2014b. We estimated both *θ* and the Mahalanobis distances for wing shape, the orange spot, and genital shape, within species (using allopatric populations) and among species (using the individuals of either species that were collected from the eastern‐ or westernmost part of the contact zone). Given the much denser sampling, we excluded individuals closer to the secondary contact zone. We subsequently compared *θ* and the Mahalanobis distances within and among species using an ANOVA with a Tukey's HSD post hoc test.

### PHENOTYPIC DIFFERENTIATION ALONG THE CONTACT ZONE

We then tested whether the phenotypic traits of *E. cassioides* and *E. tyndarus* become more dissimilar the closer they are in proximity, which could indicate character displacement. For this, we repeated the PC analysis, including only individuals from across the contact zone to constrain the morphospace to phenotypic differentiation upon secondary contact, as PCs based on the full dataset could be driven by interspecific differentiation between allopatric populations. We then analyzed the scores of PC1 for wing shape, orange spot, and genital shape, as well as for all individual traits, by fitting linear models. Explanatory variables were the *distance* of each specimen from the westernmost individual (in km), the *species* (*E. cassioides* or *E. tyndarus*), and their interaction. We subsequently repeated the analysis by fitting the same linear models for each morphological trait separately, also applying a false discovery rate (Benjamini and Hochberg 1995) correction to account for multiple testing.

Next, we fitted simple sigmoid clines across all individuals from the contact zones separately for PC scores of wing shape, orange spot, and genital shape for PC1–PC4. Cline estimations, using maximum likelihood approximation (bbmle package in r; Bolker et al., 2017), were based on the equations of Derryberry et al. (2014) adapted from Westram et al. (2018) to allow for an individual‐based analysis. We fitted the clines using the individual geographic distances (km) from the westernmost individual. The best model was selected using Akaike's information criterion. We also performed a cline analysis for the genomic PC1 axis, for which hybrid individuals were included.

Finally, to test if the intraspecific **P** matrix may change along the contact zone, we employed an overlapping sliding‐window approach, where for each species, we took 30 individuals starting from the point of contact and estimated *θ* and the pairwise Mahalanobis distance between these samples and individuals from the utmost western‐ (for *E. cassioides*) or easternmost (for *E. tyndarus*) part of the contact zone. Window step size was by 10 individuals, that is, removing the 10 individuals closer to the point of contact and including the following 10 individuals closer to the respective western‐ or easternmost parts of the range. We estimated the significance of *θ* and the Mahalanobis distances with 10,000 bootstrap replicates. Because we included individuals that were not genotyped but assigned to a species by the LDA, we performed an additional local PCA for each species and phenotypic dataset and removed individuals outside the 95% CI from the subsequent **P** matrix analyses. We tested for changes in the **P** matrix along the contact zone for each phenotypic dataset using linear mixed‐effect models. The response variable was *θ* or the Mahalanobis distance, with the fixed effect being the interaction between *distance* (the average position along the contact zone in km from west to east) and *species*. The random effect was the state of statistical significance estimated by our bootstrap approach.

### DIFFERENTIATION IN THE ABIOTIC ENVIRONMENT

To assess the potential for niche differentiation in the abiotic environment between *E. cassioides* and *E. tyndarus*, we first tested whether the multivariate habitat would differ between the species among allopatric populations and between allopatric populations and the contact zone. As such, we summarized the environmental parameters in a PC analysis, and used the PC scores of the leading axis to fit a linear mixed‐effects model with *species* and *population type* (i.e., allopatric or contact zone) as explanatory variables and location as a random effect. We estimated the significance of each model with a type II Wald *χ* ^2^ test. We further selected the seven least‐correlated, ecologically meaningful variables based on Pearson's correlation coefficient and variance inflation factor: potential evapotranspiration (mm/day) in July (EvapoJul), isothermality (Isothermal), precipitation seasonality (PrecSeason), precipitation in September in mm (PrecSept), mean temperature of the wettest quarter (TMeanWetQ), the minimum temperature in August (TMinAug), and temperature seasonality (TSeason). Based on these variables, we ran niche similarity tests with 1000 replications using the *ecospat* package (Di Cola et al. 2017) in R to quantify niche overlap (Broennimann et al. 2012) based on Warren's *I* (Warren et al. 2008) and Schoener's *D* (Schoener 1968) between *E. cassioides* and *E. tyndarus* in the allopatric and the contact zone and to determine if the species may undergo niche divergence.

We then focused on differences across the contact zone by fitting a linear model based on PC1 scores for contact zone individuals only, with *distance* from the westernmost individual and *species* as explanatory variables. We extracted the variance components of this model to disentangle each abiotic variable's contribution and then fitted individual sigmoid clines for the seven focus variables across the contact zone as for the phenotypic clines. We similarly compared substrate classes among species.

## Results

### GENOMIC STRUCTURE

Consistent with former studies (Gratton et al. 2016; Lucek et al. 2020), the genomic PC1 accounted for 70.2% of the total variation and clearly separated the two focal species, both between allopatric populations and along the contact zone (Fig. 1). We further identified three putative F1 hybrids that were genetically and phenotypically intermediate between *E. cassioides* and *E. tyndarus*, and found no apparent backcrossing (Figs. 1, S3). ADMIXTURE similarly separated the two species (Fig. 1c).

### PHENOTYPIC DIFFERENTIATION BETWEEN ALLOPATRIC SITES

Of 830 specimens, 811 were retained as being within the 97% CI, with 464 samples coming from the contact zone. Across the contact zone, we counted 186 *E. cassioides* (*N*_Genotyped_ = 71; *N*_Assigned_ = 115) and 278 *E. tyndarus* (*N*_Genotyped_ = 115; *N*_Assigned_ = 163).

**Figure 2:**
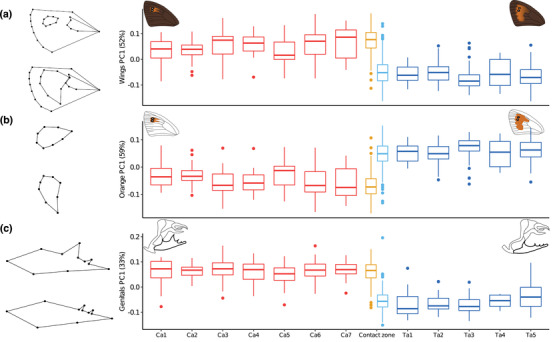
Boxplots representing the phenotypic variation among populations along the leading principal component axes (PC1) for (a) wing shape, (b) the shape of the orange spot, and (c) genital shape. Wireframes depict phenotypes at a score of 0.1 and –0.1, respectively. Populations for each species are arranged from west to east. The contact zone is separated by species. Colors indicate species and location: red = allopatric *Erebia cassioides*, orange = contact zone *Erebia cassioides*, light blue = contact zone *Erebia tyndarus*, dark blue = allopatric *Erebia tyndarus*. Pictograms depict representative morphologies of each species (following Sonderegger 2005).

Across all samples, we found wing shape to be significantly different between species along PC1, accounting for the majority of phenotypic variation (52.2%; *χ* ^2^_1,811_ = 861.64, *P* < 0.001, Fig. 2a). Here, *E. cassioides* and *E. tyndarus* differ by the shape and extent of the orange spot on the forewing, which in *E. cassioides* is generally smaller, does not extend as far downward toward the anal margin of the wing, and often does not reach the cell of the wing (Table S2; Fig. 2a). Wing shape did not differ within species between individuals from the contact zone and allopatric sites (*χ* ^2^_1,811_ = 2.39, *P* = 0.122). Focusing on the orange spot only, we observed the same pattern (Fig. 2b), that is, a marked difference between species along PC1, explaining a majority of phenotypic variation (59.3%; *χ* ^2^_1,811_ = 874.97, *P* < 0.001), but no difference between individuals from the contact zone and allopatric sites (*χ* ^2^_1,811_ = 2.46, *P* = 0.117). The shape of the male genitalia, a key character in distinguishing between *E. cassioides* and *E. tyndarus* (Lorkovic 1958; Sonderegger 2005), differed strongly between the species (*χ* ^2^_1,811_ = 1405.29, *P* < 0.001; Fig. 2c) along PC1 (32.5%). Overall, *E. cassioides* had shorter genital valves, and their first tooth on the valve was larger and wider than for *E. tyndarus* (Fig. 2c; Table S3). Like wing shape and the orange spot, genital shape did not differ overall between individuals from the contact zone and allopatric sites (*χ* ^2^_1,811_ = 0.14, *P* = 0.714).

**Figure 3:**
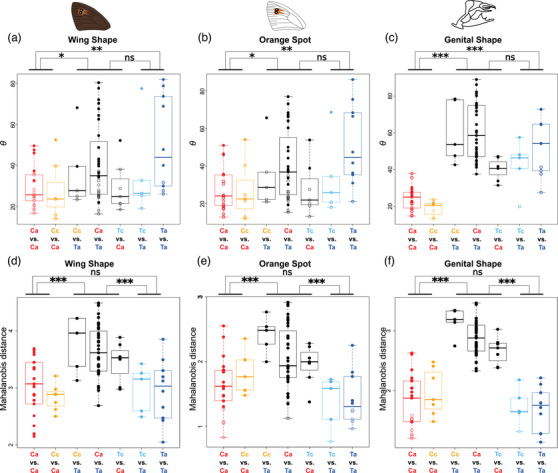
Inter‐ and intraspecific changes of population‐based **P** matrices. Boxplots summarize the angles *θ* between the population‐specific leading eigenvectors (***p***_max_) (a‐c) or the overall Mahalanobis distances between **P** matrices (d‐f) for wing shape (a and d), the shape of the orange spot (b and e), and genital shape (c and f). Filled circles indicate significant and open circles nonsignificant estimates, based on 10,000 bootstrap replicates. Population comparisons are as follows: Ca, allopatric *E. cassioides*; Cc, contact zone *E. cassioides*; Ta, allopatric *E. tyndarus*; Tc, contact zone *E. tyndarus*. *θ* for each trait category was compared, grouping intra‐ and interspecific comparisons. Significance levels based on a post hoc Tukey's HSD test: ***P < 0.001; **P < 0.01; *P < 0.05; ns, *P* > 0.05.

We also assessed differentiation within and between *E. cassioides* and *E. tyndarus* in their multivariate phenotypic covariance matrices: first, by estimating the angle *θ*, which captures the pairwise difference of the leading eigenvectors (PC1) between populations, and second, by calculating the Mahalanobis distance between population pairs, to quantify the overall differentiation between two matrices. For *θ*, we found a significant differentiation for all phenotypic trait categories (ANOVA wing shape: *F*_2,87_ = 5.23, *P* = 0.007; orange spot: *F*_2,87_ = 5.91, *P* = 0.004; genital shape: *F*_2,87_ = 56.69, *P* < 0.001; Fig. 3). Post hoc Tukey's HSD indicate that intraspecific phenotypic differentiation was significantly higher among allopatric *E. tyndarus* than allopatric *E. cassioides* (wing shape: *P* = 0.009; orange spot: *P* = 0.005; genital shape: *P* < 0.001; Fig. 3). Similarly, *θ* for interspecific comparisons was significantly higher than that for intraspecific comparisons of *E. cassioides* (wing shape: *P* = 0.047; orange spot: *P* = 0.029; genital shape: *P* < 0.001) but not of *E. tyndarus* (wing shape: *P* = 0.387; orange spot: *P* = 0.365; genital shape: *P* = 0.085). The Mahalanobis distances differed similarly for all phenotypic trait categories (ANOVA wing shape: *F*_2,87_ = 23.05, *P* < 0.001; orange spot: *F*_2,87_ = 16.93, *P* < 0.001; genital shape: *F*_2,87_ = 108.00, *P* < 0.001; Fig. 3). However, post hoc Tukey's HSD suggests no difference in intraspecific differentiation between allopatric *E. tyndarus* and *E. cassioides* (wing shape: *P* = 0.993; orange spot: *P* = 0.108; genital shape: *P* = 0.366; Fig. 3), yet all interspecific comparisons were significantly higher than the intraspecific comparisons for both *E. tyndarus* and *E. cassioides* (all *P* < 0.001). Together these results suggest that the level of intraspecific differentiation is smaller than interspecific differentiation but that the leading eigenvectors differ even among populations within a species.

### PHENOTYPIC DIFFERENTIATION ALONG THE CONTACT ZONE

**Figure 4:**
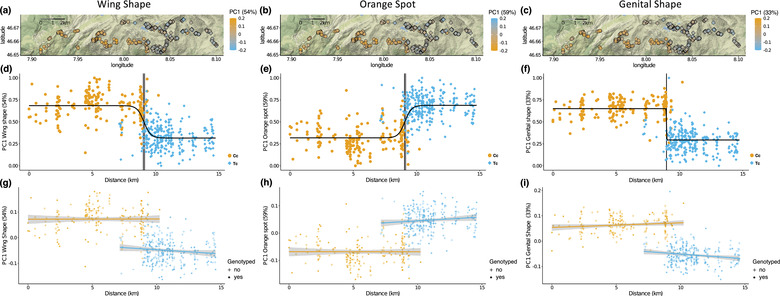
Summary of the phenotypic outcome of secondary contact. (a‐c) Elevation maps of the contact zone showing the locations of all studied *Erebia cassioides* (dots) and *Erebia tyndarus* (rhombi) individuals colored by their respective PC1 scores based on (a) wing shape, (b) shape of the orange spot, and (c) genital shape. (d‐f) Cline fits between *E. cassioides* (C in orange) and *E. tyndarus* (T in light blue) along a west‐east transect for the same traits as panels (a‐c). The black line represents the fitted cline and the vertical black bar indicates the cline center, with the gray area depicting its 95% confidence interval (CI). Each cline was fitted based on the distance (km) from the westernmost individual. PC scores were rescaled. (g‐i) Model fits based on linear models testing for an interaction between the distance from the westernmost individual and species with the respective 95% CI for each model in gray. Colors depict species (orange: *E. cassioides*; light blue: *E. tyndarus*) and symbols indicate if species were determined based on genotyping (circles) or statistically assigned to a species (crosses).

We tested if individuals sampled closer to the point of contact were phenotypically more distinct than individuals caught further away by assessing whether our phenotypic traits changed with distance across our 14.58‐km‐wide transect (Fig. 4a‐c). For wing shape, although there was a marked difference between species (linear model: *F*_1,464_ = 249.91, *P* < 0.001; Fig. 4g) along PC1 (54.3%), there was no differentiation across the transect (*distance*: *F*_1,464_ = 1.21, *P* = 0.271) for neither species (*species*×*distance*: *F*_1,464_ = 2.22, *P* = 0.137). Using the same model for each individual landmark, we similarly found that most wing traits (40 out of 46) differ between species (Table S4) but do not vary with distance across the transect (all *P* > 0.05; Table S4).

For the orange spot, results were similar to those of wing shape. The phenotypic variation in the orange spot along PC1 (59.2%) differs between *species* (*F*_1,464_ = 260.01, *P* < 0.001; Fig. 4h) but individuals closer to the point of contact did not show increased phenotypic differentiation (*distance*: *F*_1,464_ = 1.08, *P* = 0.299; *species*×*distance*: *F*_1,464_ = 1.84, *P* = 0.175). For genital shape, the same interspecific differentiation was found as for the overall dataset along PC1 (32.7%; *F*_1,464_ = 447.21, *P* < 0.001; Fig. 4i). Although *distance* was not significant (*F*_1,464_ = 0.14, *P* = 0.710), we found a significant *species*×*distance* interaction (*F*_1,464_ = 10.82, *P* = 0.001), driven by *E. tyndarus*, where genital shape shifts toward the point of contact as individuals seem to become phenotypically more similar to *E. cassioides*. The latter is reflected at the level of individual landmarks, where *distance* played no role, but interspecific differentiation occurred in 13 out of 22 landmarks and an intraspecific shift across *distance* was found for five landmarks (Table S5).

The clines for wing shape, the orange spot and genital shape all overlapped and centered around the transition between *E. cassioides* and *E. tyndarus* (Fig. 4d‐f; Table S6; wing shape: 9.08 km from the westernmost specimen [95% CI: 8.95–9.21]; orange spot: 9.10 km [95% CI: 8.99–9.21]; genital shape 8.97 km [95% CI: 8.95–8.99]). The clines were narrow compared to transect distance, that is, ranging from 35 m (genital shape), over 216 m (orange spot), to 259 m (wing shape). Clines on subsequent PC axes could only be fitted for the second PC axes for all trait categories (Fig. S4). For these, the cline centers again overlapped at around 9 km, and all were narrow (wing shape: 175 m, orange spot: 199 m, genital shape: 227 m). Likewise, the genomic cline overlapped with the phenotypic clines, as its cline center lies at 9.02 km from the westernmost individual (95% Cl: 8.96–9.08). Notably, the cline is only 125 m wide (Fig. 1d).

**Figure 5:**
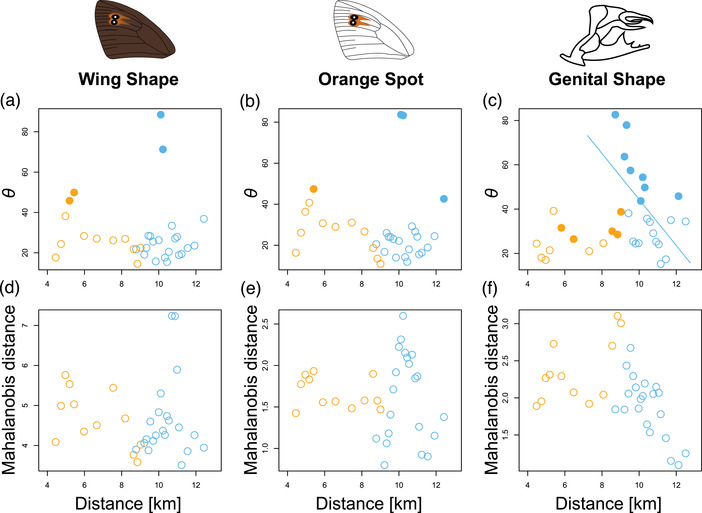
(a‐c) Intraspecific changes in the **P** matrix along the contact zone for angles *θ* between the subset specific leading eigenvectors (***p***_max_) (a‐c) or the overall Mahalanobis distances between **P** matrices (d‐f) for wing shape (a and b), the shape of the orange spot (b and e), and genital shape (c and f). The differences between the westernmost or easternmost individuals for *E. cassioides* (orange) and *E. tyndarus* (light blue), respectively, and subsets of individuals along the contact zones based on a sliding window approach are shown. Comparisons were only made within a species, where full circles depict significant and the open circles nonsignificant values based on 10,000 bootstrap replicates. The line in C depicts a significant increase in *θ* based on a linear mixed‐effect model.

Intraspecific changes in the **P** matrix also occurred along the contact zone for both species (Fig. 5). For *θ*, these changes often involved several phenotypic changes, as indicated by differences in the trait loadings of the local leading PC axes compared to individuals from the eastern or westernmost part of the contact zone (Tables S7–S9). For *E. tyndarus*, differences in *θ* occurred primarily close to the contact zone, but an association with the distance gradient only occurred for genital shape as indicated by an overall *species*×*distance* interaction (*χ* ^2^_1_ = 11.72, *P* < 0.001; Fig. 5c). Here, intraspecific phenotypic changes primarily occurred along the horizontal axes, where *E. tyndarus* showed shorter valves near the contact zone than further away (Table S9). For *E. cassioides*, *θ* differed only in some cases for wing shape and the orange spot, more closely to the western part of the contact zone (Fig. 5). Although the overall **P** matrix similarly varied along the contact zone based on Mahalanobis distances, none of these comparisons were significant.

### ABIOTIC ENVIRONMENT

**Figure 6:**
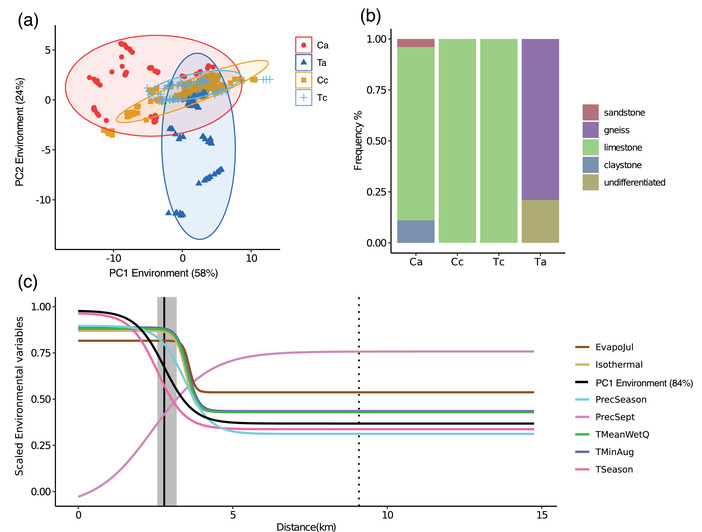
Summary of the abiotic environment. (a) Biplot of the two leading principal component axes based on all environmental variables (red = allopatric *E. cassioides* [Ca], orange = contact zone *E. cassioides* [Cc], light blue = contact zone *E. tyndarus* [Cc], dark blue = allopatric *E. tyndarus* [Ta]). (b) Bar plots summarizing the geological substrate classes where allopatric individuals and individuals from the contact zone were collected. (c) Cline fits between *E. cassioides* and *E. tyndarus* along a west‐east transect for the seven least correlated environmental variables: EvapoJul = potential evapotranspiration (mm/day) in July, Isothermal = isothermality, PrecSeason = precipitation seasonality, PrecSept = precipitation in September in mm, TMeanWetQ = mean temperature of the wettest quarter, TMinAug = minimum temperature in August, TSeason = temperature seasonality, and for PC1 based on all environmental variables = PC1 Environment (84%). The full vertical black bar indicates the cline center for PC1 environment, with the gray area depicting its 95% confidence interval. The dotted vertical line indicates the cline center for PC1 of genital morphology (see Fig. 4f). All cline fits were performed on scaled values (see *Materials and Methods*).

Allopatric *E. cassioides* and *E. tyndarus* differed significantly in their abiotic environment along PC1 (*χ* ^2^_1,811_ = 9.64, *P* = 0.002; Fig. 6a), which accounts for 58.1% of the total variation and is mainly driven by temperature‐related variables, growing degree days and some precipitation‐related variables (Table S10). Although individuals from the contact zone and the allopatric sites did not differ in their abiotic environment (*χ* ^2^_1,811_ = 0.43, *P* = 0.512), there is some differentiation between species (*χ* ^2^_1,811_ = 4.30, *P* = 0.038). Niche similarity based on the seven least correlated variables showed limited overlap between allopatric *E. cassioides* and *E. tyndarus* (Ca vs. Ta: Warren's *I* = 0.10, Schoener's *D* = 0.07), whereas the overlap was higher at the contact zone (Cc vs. Tc: *I* = 0.50, *D* = 0.30). The niche of allopatric individuals and individuals at the contact zone was more similar for *E. cassioides* (Ca vs. Cc: *I* = 0.38, *D* = 0.23) than *E. tyndarus* (Ta vs. Tc: *I* = 0.02, *D* = 0.08). Randomized replications suggest that the compared niches were not more diverged than expected by chance (all *P* > 0.05). In addition, for allopatric individuals, the two species occurred on different substrates, where *E. cassioides* is found primarily on limestone and *E. tyndarus* on gneiss substrates (Fig. S5). In contrast, individuals along the contact zone were collected exclusively on limestone, independent of species (Figs. 6b, S5).

In the contact zone, the abiotic environment changed with *distance* across the transect (*F*_1,464_ = 25.52, *P* < 0.001), and differed between and within *species* (*F*_1,464_ = 4.44, *P* = 0.036; *species*×*distance*: *F*_1,464_ = 339.40, *P* < 0.001). At the level of individual ecological variables, differences most commonly include changes across the transect within one species (*E. cassioides*), whereas interspecific differentiation was found for only nine variables related to temperate and precipitation (Table S11). This shift within *E. cassioides* was reflected in the clines of the environmental variables, which were shifted westward compared to the phenotypic and genomic clines (Fig. 6c), indicating that the abiotic environmental variables are not correlated with the position of the contact zone. For PC1, the cline center lies 2.82 km east of the westernmost individual (95% CI: 2.54–3.11 km) and the cline centers for each of the seven least‐correlated variables overlap at about 3.5 km (Table S12). These results suggest a transition in the abiotic environment within the *E. cassioides* habitat from a slightly warmer to a colder environment with higher isothermality and potential evapotranspiration, but lower precipitation.

## Discussion

Secondary contact may trigger reinforcement of existing or additional barriers to gene flow within a contact zone, but to which degree reinforcement occurs in nature or contributes to speciation remains unclear (Kulmuni et al. 2020; Matute and Cooper 2021). A final stage of speciation is coexistence and widespread sympatry, which may not always be achieved, for example, when species or lineages fail to evolve enough ecological differentiation (Tobias et al. 2020; but see M'Gonigle et al. 2012) or when hybridization occurs even under high assortative mating (Irwin and Schluter 2022). As such, secondary contact zones may differ from one another in where they could be placed within the “gray zone” of late‐stage speciation (Roux et al. 2016; Burbrink et al., 2021). Here, we studied the inter‐ and intraspecific phenotypic and environmental variation of two closely related *Erebia* butterfly species that form a very narrow contact zone in the Swiss Alps and rarely hybridize (Descimon and Mallet 2009; Gratton et al. 2016; Lucek et al., 2020; Fig. 1).

### THE GEOGRAPHIC EXTENT OF SECONDARY CONTACT

Contact zones in butterflies may extend over tens to hundreds of kilometers, often with substantial gene flow between lineages as found in *Lycaeides* butterflies from North America (Gompert et al. 2010) or tropical *Heliconius* butterflies (van Belleghem et al. 2021). In contrast, the closely related species *E. cassioides* and *E. tyndarus* are a case of exceptionally limited geographical contact, with their contact zone being less than a kilometer wide (Lucek et al. 2020; Figs. 1, 4). However, an important gap in the aforementioned study was that it used only few individuals and focused on the narrow geographic region where the two species meet, precluding any inference about the full geographic scale of secondary contact and the potential outcome of reinforcing selection. When we expanded the sampled range for both species to several allopatric sites across the Swiss Alps, we found significant interspecific genomic differentiation with few intermediates (Figs. 1, S3), as well as differentiation for all phenotypic trait categories both for the respective leading axes of phenotypic variation (Fig. 2) and for the population‐based multivariate covariance (**P**) matrices (Fig. 3). The **P** matrices further indicate that intraspecific differentiation is more predominant across allopatric *E. tyndarus* populations for the leading eigenvectors, especially for wing shape‐related traits (Fig. 3a,b).

Likewise, genital shape showed little intraspecific differentiation for *E. cassioides*, compared to *E. tyndarus*, especially along the leading axis (Fig. 3c), suggesting stronger genetic constraints for these traits in the former species. Notably, at the broad scale, we did not find a statistical difference between allopatric and contact zone individuals, suggesting that putative ongoing reinforcement may only be at play across our studied contact zone. Indeed, our studied transect comprising the contact zone is relatively wide (14.58 km) compared to the actual point of contact (Fig. 4). As dispersal capabilities of *Erebia* butterflies are often limited, that is, a few hundred meters (Polic et al. 2014), individuals at the extreme end of this transect may experience only little to no interspecific contact, limiting the spread of potential reinforcing selection.

Given the consistent interspecific phenotypic differentiation among allopatric sites, we subsequently focused on the contact zone to assess the potential for reinforcement by fitting clines. Theory implies that the cline width and slope for a given trait depend on the extent of gene flow and the strength of selection against hybrids in a contact zone (Bímová et al., 2011; Bewick and Dyer, 2014). A steep cline may result from strong selection against genetically or phenotypically intermediate individuals, suggesting that reinforcement could be at play (May et al. 1975; Bewick and Dyer, 2014). If clines of different traits overlap, it may indicate that they are coupled, either because the different reproductive barriers may be genetically linked or because the strength and type of selection acting on them are the same (Slatkin 1975; Kruuk et al. 1999; Bierne et al. 2011; Bewick and Dyer, 2014). The genomic cline and the clines for wing shape, orange spot, and genital shape across the contact zone were extremely steep and narrow, with estimated widths for the cline center ranging between 35 and 259 m (Figs. 4, S4). Therefore, direct or indirect selection against intermediate phenotypes for these traits seems likely, especially for genital shape, given the abrupt phenotypic transition (Fig. 4f). Notably, the phenotypic clines spatially overlap within this very narrow zone despite a putative lack of apparent geographic or environmental barriers at that location (Fig. 6b,c), indicating that they may experience a common selection regime. The phenotypic and genomic clines could otherwise be a by‐product of interspecific differentiation in the absence of heterospecific mating, yet hybridization occurs at this narrow point of contact. In contrast, most of the clines for the abiotic environment are much smoother and wider, and none overlap with the phenotypic clines (Fig. 6c), suggesting that ecological differences do not primarily drive the phenotypic clines.

### REINFORCEMENT—OR THE LACK THEREOF

Given the narrow phenotypic clines, we tested for the “classic” signal of reinforcement, that is, whether the phenotypic traits become more dissimilar close to the point of contact when compared to allopatry (Coyne and Orr 2004; Servedio 2009). Importantly, this is only an indirect estimation, as character displacement across a contact zone may be consistent with reinforcement, but does not provide a direct measure of the presence, strength, or impact of this process. Reinforcement has been suggested to act on wing shape and color patterns in butterflies when involved in mate choice, as in the genus *Agrodiaetus* (e.g., Lukhtanov et al., 2005) or mimetic *Heliconius* butterflies (Jiggins et al. 2001; Kronforst et al. 2007). For our *Erebia* contact zone, we found limited intra‐ but substantial interspecific variation in wing shape with no statistical support for potential ongoing reinforcement (Fig. 4g,h).

The phylogenetic splits between many butterfly species that currently form secondary contact zones across Europe are relatively old, as they predate several glaciation cycles (Ebdon et al. 2021). Such species may consequently have already been in contact during the Pleistocene and potentially experienced reinforcing selection in the past, as could be the case for *E. cassioides* and *E. tyndarus*, whose split has similarly been proposed to predate the last glaciation (Peña et al. 2015). Reinforcing selection could therefore have occurred during a past interglacial period and phenotypes subsequently fixed in different glacial refugia. This could have caused the observed limited level of intraspecific differentiation, especially within *E. cassioides*. Reinforcement could also have occurred more recently, as the footprint of reinforcement is expected to diminish when intermediates become rarer, especially if the trait on which reinforcement acts has a weak or intermediate effect on isolation (Bank et al. 2012). In such cases, the completion of speciation requires additional factors, which in our case may include differential infection by *Wolbachia* (Telschow et al. 2007; Lucek et al. 2020). If the traits under reinforcement caused strong isolation, then reinforcement alone can suffice to complete speciation (Bank et al. 2012), which in the case of *E. cassioides* and *E. tyndarus* seems not to have happened.

Unlike wing shape, the genital shape did show evidence for intraspecific phenotypic differentiation for PC1 along the contact zone for *E. tyndarus*, whose individuals close to the point of contact differed significantly in their genital shape when compared to individuals from the eastern part of the transect (Fig. 4i). Surprisingly, individuals at the point of contact tend to become more similar to *E. cassioides* (Fig. 4i). Phenotypic convergence in sympatry can result from interspecific competition for essential resources, territoriality, or behavioral signals (Cody 1973; Leary 2001; Reifová et al. 2011). Although increased convergence is contrary to classic predictions of reinforcement, different scenarios may account for the observed patterns: First, genital morphology may have resulted from introgressive hybridization (Reifová et al. 2011). However, this seems unlikely given the apparent low hybridization rates (Lucek et al. 2020; Fig. 1) and the narrow genomic (Fig. 1d) and phenotypic clines (Fig. 4). Second, intrinsic genetic incompatibilities may have evolved in allopatry in one or both species, preventing interspecific gene flow upon secondary contact. We could then still expect selection toward increased differentiation in mate choice‐related traits to avoid interbreeding, unless assortative mating also arose as a by‐product (Kulmuni and Westram, 2017). In such a scenario, reproductive interference could be at play, that is, interspecific mating interactions leading to negative fitness effects on either one or both species, often resulting from incomplete species recognition. Much like reinforcement, reproductive interference can lead to a pattern of RCD (Gröning and Hochkirch 2008). The key difference with reinforcement is that under reproductive interference, selection may act directly on phenotypes related to mating behavior to enhance preference for heterospecific signals (Shaw and Mendelson 2013). Reproductive interference may operate even when there is no interspecific gene flow upon secondary contact, except perhaps for a few F1 hybrids that do not backcross (Hollander et al., 2018). Although the presence of only F1 hybrids with no further gene flow in our system (Fig. 1d) is consistent with reproductive interference, a clear distinction between reproductive interference (direct selection due to wasteful mating interactions) and reinforcement (indirect natural selection to avoid unfit hybrids and a gradual reduction in gene flow) would require to test for past gene flow (Hollander et al., 2018). Furthermore, both reinforcement and reproductive interference could result in RCD, and would require increased differentiation in traits associated with mate choice, which does not seem to involve the traits we assessed for wing shape (Figs. 2, 4). However, prezygotic species recognition may, in this case, involve additional characters, such as olfactory cues through chemical signaling, which are often involved in mate choice in butterflies (Andersson et al. 2007; Constanzo and Monteiro 2007; Li et al. 2017). Although we could not assess the possibility of reinforcement of chemical signals between *E. cassioides* and *E. tyndarus*, this could be a promising avenue for future research in this system. Similarly, three‐dimensional microimaging of genital morphology could provide further insights in this system.

Even if closely related species are phenotypically strongly differentiated, they may evolve along shared evolutionary trajectories, whereby the leading eigenvectors of the species‐specific **P** matrices (***p***_max_) would align (Dochtermann and Matocq 2016). Conversely, our intra‐ and interspecific comparisons between allopatric populations suggest that ***p***_max_ differ between *E. cassioides* and *E. tyndarus*, especially for genital shape (Fig. 3). The difference between wing shape and genital shape could result from a higher genetic integration of these traits (Arnold et al. 2008). *Erebia tyndarus* shows a high level of intraspecific phenotypic differentiation for wing shape, which could indicate a higher standing genetic variation for these traits or additional intraspecific differentiation (Eroukhmanoff and Svensson 2011). Similar to individual traits, the intraspecific **P** matrix may change along a contact zone (Dochtermann and Matocq 2016). Based on our sliding window approach, intraspecific changes occasionally occur for both species along the contact zone for ***p***_max_ but not necessarily for the overall **P** matrix (Fig. 5). The latter was especially true for *E. tyndarus* individuals spatially close to the contact zone; however, to which degree these patterns could reflect local responses to selection requires further investigation. Although it has been suggested that the **G** and **P** matrix estimation may require large sample sizes (Melo et al. 2015), Eroukhmanoff and Svensson (2011) suggest that small sample sizes are more likely to result in increased similarity. As such, our estimates are probably on the more conservative side. Overall, our analyses implicate that *E. tyndarus* is likely less phenotypically constrained than *E. cassioides*, as indicated by its increased level of intraspecific differentiation both across allopatric populations and along the contact zone (Figs. 3, 5).

Despite significant phenotypic differentiation in traits linked to mate choice in other butterfly systems (e.g., Kemp and Rutowski 2011; Hinojosa et al. 2020), our focal species fail to coexist. In addition, the presence (*E. cassioides*) or absence (*E. tyndarus*) of *Wolbachia* (Lucek et al. 2021, 2020), which may act as an intrinsic postzygotic barrier between the two species, nonetheless does not seem to prevent interspecific gene flow. Temporal isolation also seems unlikely to cause strong isolation, given that both species fly together at the contact zone (Figs. S1, S3). A common requirement for spatial coexistence is the utilization of different ecological niches (Leibold and McPeek 2006), although other factors such as sexual selection can similarly promote coexistence on their own (M'Gonigle et al. 2012). Conversely, even a very small amount of hybridization or just interbreeding itself between lineages with strong assortative mating could suffice to prevent coexistence (Irwin and Schluter 2022). However, if neither ecology, sexual selection, nor their interaction suffices to complete reproductive isolation, competing species may stay in stable parapatry at contact zones (Tobias et al. 2020). Co‐occurring *Erebia* species have been shown to differ in their microhabitat use (Kleckova et al. 2014), but to which degree this may be the case for our studied species remains unknown. Both *E. cassioides* and *E. tyndarus* use *Festuca* sp. grasses as their larval host plants (Sonderegger 2005), and these occur in abundance in the contact zone (pers. obs.). However, it is not known whether they share the same host plant species. Similarly, there is limited evidence that nectar plants are shared by adults (Sonderegger 2005).

Focusing on more broad‐scale ecological data, we found that the niches of the two species differ between their allopatric populations, but become more similar at the contact zone; however, there is no indication of niche divergence among species. Interestingly, *E. tyndarus* from the contact zone seem to occupy a different niche than their allopatric counterparts (Fig. 6a). The latter is especially true for the geological substrate, where both species occur on limestone along the contact zone, whereas allopatric *E. tyndarus* primarily occur on gneiss (Fig. S5). The geological substrate is a commonly used proxy to describe species distributions of Alpine butterflies (Illán et al. 2010), including *Erebia* (Sonderegger 2005). Different substrates may have different effects on caterpillars in terms of temperature, humidity, and presence of fungal endophytes, even when host plants are otherwise the same (Johnson et al. 1968). However, which aspects of the environment may be causal in shaping the actual distributions of our *Erebia* is unknown. Our result suggests that *E. tyndarus* may be able to expand its niche to different geological substrates, whereas this seems less likely for *E. cassioides*.

Focusing on the zone of secondary contact, we did not detect a signal of reinforcement linked to ecology, as the occupied habitat appears not to be significantly differentiated between the two focal species at the contact zone. Indeed, niche overlap between the species was highest there, and the cline in the abiotic environment does not overlap with the phenotypic clines (Fig. 6c). Therefore, it may be possible that both species are more generalistic in their habitat use, as has previously been found in butterflies (e.g., Vodă et al., 2015), or that they are genetically constrained and cannot occupy different microhabitats. To which degree this could have contributed to the lack of coexistence requires further experimental investigation, and assessing the potential difference in microhabitats may be especially promising.

## Conclusion

Given their extremely narrow contact zone together with the limited level of interspecific gene flow, *E. cassioides* and *E. tyndarus* fall within the gray zone of advanced or late‐stage‐speciation (Roux et al. 2016; Kulmuni et al. 2020). However, speciation is not complete as the species fail to coexist. Given the scarcity of hybrids, other prezygotic barriers are likely at play, but to which degree they, and the apparent lack of ecological niche divergence, could have contributed to the formation of a secondary contact zone that has been stable for decades (Warren 1954; Sonderegger 2005) requires further investigation. Interestingly, we did not find strong evidence for current reinforcement of our studied traits. Given that the split between the two species could be old, we may observe the outcome of repeated secondary contact following past reinforcement. The above suggests that the *cassioides‐tyndarus* system provides an intriguing case of nearly complete speciation, allowing to study the interplay between selection and ecology on the formation of barriers to gene flow and species coexistence. Similar processes may be more commonly at play among alpine species, where closely related species either form zones of secondary contact or exclude each other (e.g., Descimon and Mallet 2009; Capblancq et al. 2020).

## AUTHOR CONTRIBUTIONS

HA and KL conceived and developed the study. HA collected and analyzed the phenotypic and genomic data. TP extracted and analyzed the ecological data. HA wrote the manuscript with input from KL and TP.

## CONFLICT OF INTEREST

The authors declare no conflict of interest.

## DATA ARCHIVING

All phenotypic data and codes are deposited at Zenodo (https://doi.org/10.5281/zenodo.6961887 at https://zenodo.org/record/6961887#.YxCvn‐xBwyk). Due to the file sizes, the genomic data are deposited in two sections at Zenodo: part 1 (123 samples) at https://doi.org/10.5281/zenodo.7041396 (https://zenodo.org/record/7041396#.YxJVhuxBwyk) and part 2 (112 samples) at https://doi.org/10.5281/zenodo.7041545 (https://zenodo.org/record/7041545#.YxIryOxBwyk).

Associate Editor: C. Wagner

Handling Editor: M. Zelditch

## Supporting information


**Figure S1**: Boxplots representing the days at which individuals of E. cassioides (C) and E. tyndarus (T) were sampled in our study for A) all allopatric samples and B) individuals from the contact zone
**Figure S2**: Landmark placement for geometric morphometric analysis. Yellow and orange dots represent the location of landmarks on scanned images
**Figure S3**: Phenotypes of hybrids between *Erebia cassioides* and *Erebia tyndarus*.
**Figure S4**: Summary of the PCA across the contact zone and the outcome of secondary contact for the first four PC axes for each phenotypic category.
**Figure S5**: Geological substrates in the study area in the Alps projected in ArcGIS v.9.3.1.

Supplementary Table: S1‐S12
