# Supplementary material for: Secondary contact rather than coexistence—Erebia butterflies in the Alps
Source: Evolution. 2022 Oct 5;76(11):2669–86. doi: 10.1111/evo.14615 (PMC9828779; doi:10.1111/evo.14615)
Supplement: Supplementary file 1 — Figure S1: Boxplots representing the days at which individuals of E. cassioides (C) and E. tyndarus (T) were sampled in our study for A) all allopatric samples and B) individuals from the contact zone Figure S2: Landmark placement for geometric morphometric analysis. Yellow and orange dots represent the location of landmarks on scanned images Figure S3: Phenotypes of hybrids between Erebia cassioides and Erebia tyndarus. Figure S4: Summary of the PCA across the contact zone and the outcome of secondary contact for the first four PC axes for each phenotypic category. Figure S5: Geological substrates in the study area in the Alps projected in ArcGIS v.9.3.1. [file EVO-76-2669-s001.docx]

SUPPLEMENTARY FIGURES:

Figure S1: Boxplots representing the days at which individuals of E. cassioides (C) and E. tyndarus (T) were sampled in our study for A) all allopatric samples and B) individuals from the contact zone. For the contact zone, three putative F_1_ hybrids (H) are represented in light green.


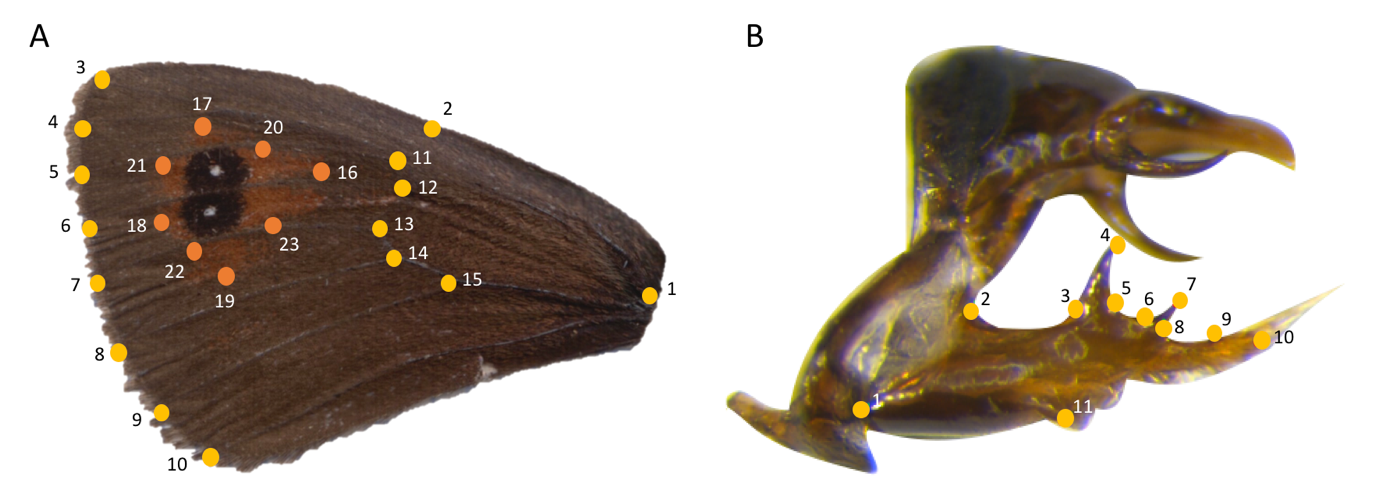


Figure S2: Landmark placement for geometric morphometric analysis. Yellow and orange dots represent the location of landmarks on scanned images. A. an Erebia dorsal forewing, where landmarks 1-15 are related to outline and wing venation. LM1: base of the wing, LM2: outer margin of subcosta 1, LM3: outer margin of radius 4, LM4: outer margin of radius 5, LM5,6,7: outer margins of media 1,2,3 respectively, LM8: outer margin of cubitus A1, LM9: outer margin of cubitus A2, LM10: outer margin of anal veins A1 + A2, LM11: intersection of cell and media 1, LM12: intersection of cell and media 2, LM13: intersection of cell and media 3, LM14: intersection of cell and cubitus A1, LM15: intersection of cell and cubitus A2. Landmarks 16-23 (in orange) represent the orange spot: LM16: basal extent of orange spot, LM17: upper extent of orange spot, LM18: outer marginal extent of orange spot, LM19: lower extent of orange spot, LM20: edge of orange spot at the midpoint between LM16 and 17, LM21: edge of orange spot at the midpoint between LM17 and 18, LM22: edge of orange spot at the midpoint between LM18 and 19, LM23: edge of orange spot at the midpoint between LM19 and 16. B. photograph of the male Erebia genital apparatus, with landmarks placed to trace the outline of the right genital valve. LM1: basal extent of genital valve, LM2: upper extent of the valve base, LM3: innermost base of the first valve tooth, LM4: apex of first valve tooth, LM5: outermost base of the first valve tooth, LM6: innermost base of the second valve tooth, LM7: apex of second valve tooth, LM8: outermost base of the second valve tooth, LM9: apex of third valve tooth, LM10: cucullus (apex of the valve), LM11: lowest extent of the valve, underneath first tooth.

Figure S3: Phenotypes of hybrids between Erebia cassioides and Erebia tyndarus. A-C) Genital and wing morphology of the three hybrid individuals found in our dataset. For individual B no genital morphology could be established. D.) Map of the contact zone focusing on the exact point of contact between E. cassioides (Cc in orange) and E. tyndarus (Tc in light blue), with the three hybrid individuals marked in green.

Figure S4: Summary of the PCA across the contact zone and the outcome of secondary contact for the first four PC axes for each phenotypic category. A- C. Screeplots of the PCAs of wing shape, orange spot, genital shape respectively. The first four axes account for 80.6% of total variation for wing shape, 90.9% for the orange spot, and 74.2% for genital shape respectively. A,B,C 1-4) PC scores of the first 4 axes, of E. cassioides (orange) and E. tyndarus (blue) along a west-east transect in the contact zone for A1-4) wing shape, B1-4) orange spot and C1-4) genital shape. For each phenotypic trait, clines could be fitted for the first two axes (A1-2, B1-2, C1-2). For these, the black line represents the fitted cline, the vertical black bar indicates the cline centre, with the grey area depicting its 95% confidence interval. Each cline was fitted based on the distance (km) from the westernmost individual. PC scores were rescaled.

Figure S5: Geological substrates in the study area in the Alp projected in ArcGIS v.9.3.1. Subtrates extracted from EuroGeoSurvey’s European Geological Data Infrastructure (EGDI; Tulstrup et al., 2016). Predominant rock types are represented by their respective colour overlays. Locations of Erebia samples are denoted by red dots. Ca= Erebia cassioides allopatric, Cc = E. cassioides contact zone, Tc = Erebia tyndarus contact zone, Ta = E. tyndarus allopatric.
